# Smartphone applications targeting borderline personality disorder symptoms: a systematic review and meta-analysis

**DOI:** 10.1186/s40479-020-00127-5

**Published:** 2020-06-16

**Authors:** Gabrielle S. Ilagan, Evan A. Iliakis, Chelsey R. Wilks, Ipsit V. Vahia, Lois W. Choi-Kain

**Affiliations:** 1 McLean Hospital, 115 Mill St, Belmont, MA 02478 USA; 2 Harvard University, Cambridge, USA; 3 Harvard Medical School, Boston, USA

**Keywords:** Smartphone applications, Borderline personality disorder, eMental health, Suicide

## Abstract

**Background:**

Smartphone applications could improve symptoms of borderline personality disorder (BPD) in a scalable and resource-efficient manner in the context limited access to specialized care.

**Objective:**

This systematic review and meta-analysis aims to evaluate the effectiveness of applications designed as treatment interventions for adults with symptoms such as anger, suicidality, or self-harm that commonly occur in BPD.

**Data sources:**

Search terms for BPD symptoms, smartphone applications, and treatment interventions were combined on PubMed, MEDLINE, and PsycINFO from database inception to December 2019.

**Study selection:**

Controlled and uncontrolled studies of smartphone interventions for adult participants with symptoms such as anger, suicidality, or self-harm that commonly occur in BPD were included.

**Study appraisal and synthesis methods:**

*Comprehensive Meta-Analysis v3* was used to compute between-groups effect sizes in controlled designs. The primary outcome was BPD-related symptoms such as anger, suicidality, and impulsivity; and the secondary outcome was general psychopathology. An average dropout rate across interventions was computed. Study quality, target audiences, therapeutic approach and targets, effectiveness, intended use, usability metrics, availability on market, and downloads were assessed qualitatively from the papers and through internet search.

**Results:**

Twelve studies of 10 applications were included, reporting data from 408 participants. Between-groups meta-analyses of RCTs revealed no significant effect of smartphone applications above and beyond in-person treatments or a waitlist on BPD symptoms (Hedges’ *g* = − 0.066, 95% CI [−.257, .125]), nor on general psychopathology (Hedges’ *g* = 0.305, 95% CI [− 0.14, 0.75]). Across the 12 trials, dropout rates ranged from 0 to 56.7% (*M* = 22.5, 95% CI [0.15, 0.46]). A majority of interventions studied targeted emotion dysregulation and behavioral dyscontrol symptoms. Half of the applications are commercially available.

**Conclusions:**

The effects of smartphone interventions on symptoms of BPD are unclear and there is currently a lack of evidence for their effectiveness. More research is needed to build on these preliminary findings in BPD to investigate both positive and adverse effects of smartphone applications and identify the role these technologies may provide in expanding mental healthcare resources.

## Introduction

Despite the proliferation of psychological treatment of mental health disorders, about 70% of individuals in the United States in need of mental health services do not receive them [28], and the treatment gap for mental illnesses remains large internationally, ranging from 32.2% for schizophrenia to 50.2–56.3% for mood disorders to 78.1% for alcohol use and dependence (WHO [29];). While evidence-based treatments are typically delivered face-to-face by licensed professionals, the majority of those in need for treatment both in the U.S. and internationally in countries of various income levels express difficulty in accessing mental healthcare, for both attitudinal (e.g. *stigma*, low perceived need) and structural (e.g. cost, availability) reasons [1, 44]. Technology-based innovations (e.g., mental health applications) have been proposed as a way to expand the reach of psychosocial interventions and address the treatment gap [26, 27].

The treatment gap is wider still for the treatment of borderline personality disorder (BPD). In the U.S., for every mental healthcare provider trained in an evidence-based treatment for BPD, there are 5933 treatment-seeking individuals with the disorder, and this number would only rise if non-treatment-seekers were included [25]. This poses a significant public health problem since BPD is a prevalent, disabling, and potentially fatal disorder with a suicide rate of 5.9% [66] and elevated rates of physical and mental disability [20]. Individuals with BPD account for 9–20% of psychiatric emergency hospitalizations [33, 48]. Society pays a high price for the associated mortality and morbidity of BPD, with estimated costs of $12,696–19,231 per patient yearly, on the order of schizophrenia [71].

While many empirically validated treatments exist for BPD — such as dialectical behavior therapy (DBT [35];), mentalization-based treatment (MBT [3, 4];), schema-focused therapy (SFT [19];), and transference-focused psychotherapy (TFP; [8, 10]) — the intensity, specialization, and cost of these effective treatments restricts their availability [25] and their appeal to patients [36]. The limited role of medications as a definitive treatment for BPD impedes the provision of care by generalist or primary care providers based on prescribing algorithms. These intensive psychotherapies are considered a “gold standard” for BPD as they incorporate group therapy, individual sessions, consultation team meetings, and in DBT intersession skills coaching [5]. They require highly intensive training and support for practitioners, which are difficult to impossible to implement in most under-resourced locales.

Data from the National Comorbidity Survey-Replication (NCS-R [62];) demonstrate that, while individuals with BPD accessed treatments more frequently than individuals with a DSM-IV Axis I disorder, only 17% of individuals with BPD sought treatment from a psychiatrist or clinical psychologist, while 29% sought treatment from a traditional provider, i.e., a nonpsychiatric physician (18%), a social worker (4%), psychiatrist (14%), clinical psychologist (7%). Over 70% of those with BPD symptoms seeking services did so through nontraditional sources, including “spiritual advisors,” “nontraditional healers,” mental health hotlines (12%), self-help support groups (20%), internet support groups (4%), herbal medicine (8%), and consultation with “telephone psychics” (3%). Follow-up data from the National Epidemiologic Survey on Alcohol and Related Cognitions (NESARC [68];) show that 25.1% of individuals with BPD will not seek mental health treatment from a physician, therapist, or counselor in their lifetime, and that this number is higher in men (31.2%) than in women (20.6%). These numbers underscore the fact that many individuals who need BPD treatment will not access it.

Computerized interventions are one potential avenue to increase availability of components of evidence-based care to individuals with BPD who cannot access treatment, and to bolster the efficacy of existing treatments. Access to smartphones far exceeds access to mental health professionals in the U.S., with 81% of U.S. Americans now owning a smartphone [50] and 90% using the internet [51], while only about 30% of the U.S. population is able to access mental health care when they need it [28]. The evidence base for the efficacy of internet-delivered and smartphone application-mediated treatments in addressing depression, anxiety, and stress is growing [12, 34]. Smartphone applications have been utilized in the prevention and treatment of substance use disorders (for review, see [39]), schizophrenia [15], anxiety [16], and depression ([17], e.g., IntelliCare, [43]) as a means of increasing access to care. Internet-delivered interventions have similar advantages to smartphone applications, e.g. adaptability, accessibility, anonymity, flexibility in time and frequency of use [6], and have likewise been employed in treatments for a wide range of disorders [2, 22, 30, 53]. Recent years have seen an upsurge in studies of technology in the treatment of BPD, with more studies on the topic indexed on PubMed between 2013 and 2019 than in between 1984 and 2012. However, there has been no review and quantitative synthesis to date on smartphone applications targeting symptoms of BPD more broadly rather than suicidality (i.e., suicidal thoughts and behaviors) exclusively [76]. The heterogeneity of studies of smartphone interventions for symptoms of anger, suicidality, nonsuicidal self-injury (NSSI), and others that are closely associated with BPD motivates a synthesis of the evidence. The aim of this systematic review and meta-analysis is therefore to evaluate the effectiveness of smartphone applications designed as interventions for symptoms that commonly occur in adults with BPD in reducing these symptoms and general psychopathology, considering both controlled and uncontrolled designs. This report will summarize and analyze data on the population served by these smartphone applications, especially in comparison to in-person treatments; the smartphone applications’ effectiveness; therapeutic approaches and common elements; specific symptom targets; and their availability and usability.

## Methods

This systematic review and meta-analysis was conducted in accordance with PRISMA guidelines [42]. The review was not registered and no review protocol exists, since the review was not originally conducted with publication in mind and the authors wished to avoid post hoc registration.

### Identification and selection of studies

Across all available articles on MEDLINE, PsycINFO, and PubMed from database inception to December 4, 2019, we combined search terms *borderline personality disorder* and its symptoms (such as *interpersonal hypersensitivity, emptiness, affective lability,* or *self-injurious behavior*)^1^ with keywords for applications (*smartphone, smartphone application, mobile application*, or *app*) and interventions (*intervention, treatment,* or *therapy*). From this search, we identified 487 unique citations. Two authors (GI, EI) screened titles and abstracts for full-text review if they evaluated the effectiveness of treatment interventions for symptoms that are also criteria for BPD (e.g., anger, suicidality and NSSI) for adults delivered through a smartphone application, regardless of length of follow-up. Studies were included regardless of language. Even uncontrolled studies were included for pre-post comparison if they reported outcomes data on BPD-related symptoms. These criteria led to the exclusion of entries with (a) methods outside the scope of smartphone applications, (b) smartphone applications not designed specifically as interventions, (c) the absence of borderline-related symptoms as outcomes, and (d) participants below the age of 18 to ensure homogeneity of participants. To allow for a more comprehensive approach including individuals with subthreshold BPD or BPD traits, participant BPD diagnostic status was not an inclusion/exclusion criterion.

### Assessment of Bias

Two raters (EI, GI) assessed risk of bias at the study level in the RCTs using the Cochrane Collaboration Risk of Bias Assessment Tool [23], which assess bias in: adequacy of the random sequence generation; allocation concealment; blinding of participants, clinical personnel, and outcome assessors; incomplete data; and selective outcome reporting.

### Systematic review of studies

Review of the resulting smartphone applications was conducted by two investigators (EI, GI) using piloted forms and focused on identifying their (a) target audiences, (b) therapeutic approaches and targets, (c) effectiveness, and (d) intended use as adjuncts or standalone interventions. We obtained information on the availability and price of the smartphone applications, as well as the ratings (out of 5 stars) and estimated number of downloads (only available on Google Play), through the App Store and Google Play to gauge usability. We also abstracted and included in usability analysis any smartphone application usage data reported by the study authors. We computed dropout rates using data reported in CONSORT diagrams and in text.

### Meta-analysis

A between-groups meta-analysis of the RCT data was conducted. We extracted data at baseline and immediately posttreatment. Effect sizes were computed using the mean change across intervention and control conditions, and the pooled standard deviation of the differences computed using the formula (${\sigma}_{diff}=\sqrt{\sigma_1^2+{\sigma}_2^2-2\rho {\sigma}_1{\sigma}_2}$), where *σ*_1_ and *σ*_2_ are the pre- and post-standard deviations of a given arm and *ρ* is the correlation between the two measurements [59]. Positive values indicated an advantage of the intervention condition. The primary outcome was borderline-related symptoms (e.g., anger, suicidality, impulsivity). The secondary outcome was general psychopathology (psychological distress, stress, depression, overall psychopathology).

We used Comprehensive Meta-Analysis V3 to compute and pool effect sizes (Hedges’ *g*) with 95% confidence intervals, calculated using a random-effects model, with a conservative estimation of test–retest reliability of 0.7 for the included measures [58]. We assessed heterogeneity using χ^2^ and I^2^ statistics, and publication bias by visual inspection of Funnel plots, Duval and Tweedie’s Trim and Fit procedure [11], and Egger’s intercept [13]. Meta-regression analysis was used to determine if number of risk of bias criteria rated low moderated effect sizes.

## Results

**Fig. 1:**
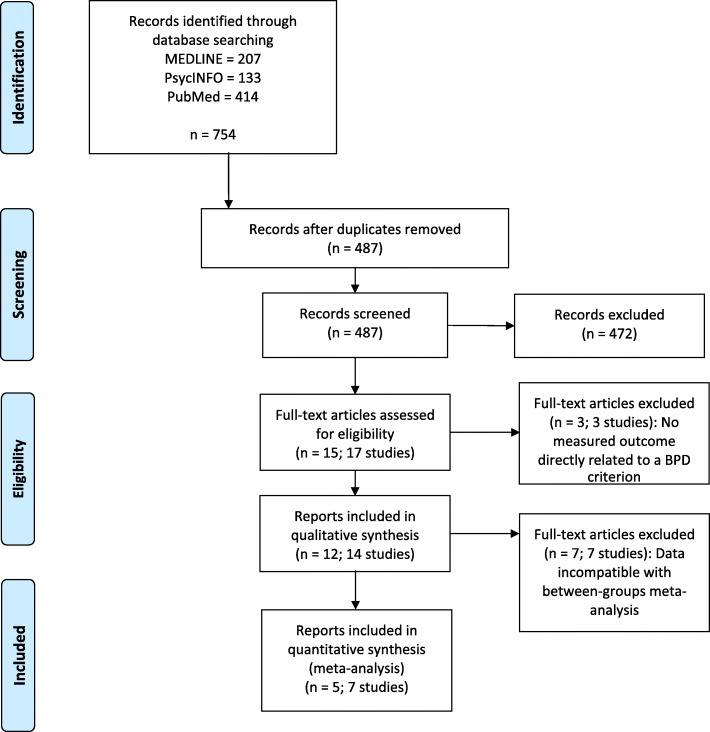
Study selection, as specified by the preferred reporting items for systematic reviews and metaanalysis (PRISMA) statement

A comprehensive literature search yielded 15 full-text articles. Of these, 3 did not measure BPD-related symptoms. A total of 12 articles describing 10 smartphone applications was included for qualitative synthesis. Of the 12 articles, 5 described 5 smartphone applications reporting data in a manner that was amenable to between-groups meta-analysis, and were included in quantitative synthesis (Fig. 1 [24]). These articles included a total of 596 participants (52.0% male; age: *M* = 32.3, 95% CI [25.5, 39.0]) from a range of recruitment settings, who used the provided smartphone application for a mean length of 6.5 weeks, 95% CI [4.8, 8.2].

Included articles studied U.S. (*N* = 6 [7, 38, 41, 45, 54, 55];), European (*N* = 3; O’Toole et al., 2019 [49, 52];), Australian (*N* = 2 [40, 67];), and international (*N* = 1 [18];) samples, although the last study had a majority of participants across articles from North America (86%) or Europe (11%). Special populations studied included veterans (*N* = 3 [7, 38, 45];), acute psychiatric inpatients (*N* = 1 [41];), and indigenous Australian individuals (*N* = 1 [67];). Only 3 studies had BPD diagnosis and DBT treatment as inclusion criteria, with Prada et al. [52] and Rizvi et al. [55] diagnosing participants using the Structured Clinical Interview for DSM–IV Axis II Disorders [14], and Rizvi et al. [54] relying on reports from DBT clinicians in the absence of any diagnostic interviews or measures. Remaining studies recruited for elevated suicidality (*N* = 5 [7, 18, 40, 47, 49];), elevated anger (*N* = 2 [38, 45];), elevated psychological distress [67], and a history of aggression or violence [41]. None assessed BPD as an outcome measure.

Notably, many of these studies had exclusion criteria driven by safety concerns and to isolate effects on the features of interest in each study. Studies excluded those with active suicidal ideation [38, 67], current primary psychotic disorder, severe depression, bipolar disorder [47], and those with alcohol or substance use disorders as well as current need for inpatient treatment [38, 47].

### Qualitative review

This investigation included review of studies of 10 different smartphone applications studied in 12 reports. The applications can be categorized in terms of symptomatic targets: (1) suicide and self-harm, (2) emotion regulation, and (3) more broad symptom targets which include both self-destructive and emotion regulation problems.

#### Suicide and self-harm applications

*TecTec* [18] aimed to decrease levels of non-suicidal self-injury by using principles of therapeutic evaluative conditioning (TEC). The application prompted users to pair positive stimuli with self-related words, and self-injurious thoughts and behaviors (SITB) stimuli with aversive stimuli in a progressively difficult game-like format that awarded points for performance. It was tested in 3 separate RCTs with similar methodologies, all of which compared the results of active TEC to a that of a control TEC in which pairings were related to neutral stimuli only, rather than aversive stimuli.

*BeyondNow* [40] tasked users with creating, editing and sharing a personalized safety plan, including contacts and emergency services. It was delivered along with Treatment as Usual (TAU) to decrease suicidality. There was no comparison condition.

*Life App’tite* [47] provided psychoeducation on suicidal thoughts, symptom and habit monitoring, a personalized safety plan, a list of places to seek help, a digital hope kit similar to the virtual hope box (VHB) of Bush et al. [7], and a library of self-help exercises (e.g. self-soothing, problem solving skills). It was compared to TAU in a Danish clinic.

*BackUp* [49] included a library of coping skills, a safety plan that included recognition of risk factors, a hope box similar to the VHB of Bush et al. [7], and a means to quickly reach out to one’s social network. It was tested on adults in the Netherlands, with no comparison condition.

#### Emotion regulation applications

*Remote Exercises for Learning Anger & Excitation Management* (RELAX [38, 45];) tracks the frequency, intensity, and cues of anger symptoms and prompts to practice personalized behavioral management exercises suggested by the smartphone application to improve anger management skills in veterans with posttraumatic stress disorder. Together with a heart rate monitor for real-time biofeedback, RELAX was used in conjunction with anger management treatment (AMT) to encourage the use of adaptive coping skills learned in treatment, and compared to AMT alone.

*EMOTEO* [52] tracked levels of aversive tension and provided audio- and videotaped mindfulness and distress tolerance exercises, chosen depending on the user’s reported level of tension. It was tested in conjunction with DBT for women with BPD, with no comparison condition.

*Headspace* [41] provides brief mindfulness meditation exercises intended to reduce anger and aggression. It was tested on patients on an acute psychiatric unit, with no comparison condition.

#### Multipurpose (suicide/self-harm, impulsivity, and emotion regulation) applications

Three smartphone applications had multiple BPD symptom targets, with 2 having suicidality or self-harm included and 2 having emotion regulation included as targets.

*Virtual Hope Box* (VHB [7];) aimed to restore emotional equilibrium and reduce suicidal ideation, provide tools for distraction, relaxation, and stress-coping; and serve as a repository of fond memories and inspirational quotes as reminders of positive life experiences, reasons for living, and people who care. Designed to be customizable, instructive in coping with negative thoughts and feelings, and useful for emotion regulation, VHB delivered in conjunction with TAU was compared to TAU enhanced by printed materials on coping with suicidal thoughts.

*DBT Coach* [54] monitored emotional intensity and urges to use substances, and encouraged labeling emotions then using the opposite action skill if they were willing to do so or evaluating the pros and cons of changing the emotion if not. With emotion-specific responses and suggestions on how to cope instead of acting impulsively, DBT Coach was implemented with adults in a DBT clinic with no comparison condition. The second trial of DBT Coach was expanded to include most DBT skills and tracked urges to self-harm [55].

*ibobbly* [67] was a suicide prevention application that tasked participants with completing 3 modules in order: first, identifying and distancing themselves from thoughts, feelings and behaviors (particularly suicidal thoughts and behaviors); next, using skills for emotion regulation; and lastly, setting goals to help them live by their values. With culturally responsive suggestions and personalized action plans, ibobbly was tested specifically in a sample of indigenous Australians compared to a waitlisted group.

Overall, in terms of study design, 5 out of the 12 articles described randomized controlled trials (RCTs [7, 18, 38, 47, 67];) while the rest were uncontrolled pre-test/post-test studies [40, 41, 45, 49, 52, 54, 55]. Franklin et al. [18] described 3 RCTs in 1 article. Three smartphone applications were designed as stand-alone interventions [18, 49, 67]. The others were delivered as adjuncts to other interventions: treatment as usual [7, 40, 47], DBT [52, 54, 55], inpatient treatment [41], and anger management treatment [38, 45]. (See Table 1 for overview.)

**Table 1:** Summary of Studies Included

Study	App Name and Description	Control Condition	Len-gth (wks)	N, % male	% with BPD	Study Inclusion Criteria	Outcomes	Drop-out^**c**^
**Suicide and Self-Harm Applications**								
[18]	**TecTec**.^a^ Positive stimuli are paired with self, and self-harm related stimuli to aversive stimuli in a gamified format	Gamified format with stimuli pairing, but only with neutral stimuli (RCT)	4	31, 19.3%	NR	Community adults with active NSSI	• ↓ in all self-injurious thoughts and behaviors (SITB) except SI in app group vs. control • ↓ in self-cutting episodes (32–40%), suicide plans (21–59%), and suicidal behaviors (33–77%)	24.4% (10)
[18]				40, 26.0%	NR			28.6% (16)
[18]				33, 41.1%	NR			47.6% (30)
[40]	**BeyondNow**. Personalized safety plan, including contacts and emergency services + Treatment as usual	None	~ 10.42	36, 33.3%	22.3%	Adults with elevated suicide risk in mental health treatment	• ↓ severity and intensity of SI • ↑ suicide-related coping	38.9% (14)
[47]	**LifeApp’tite**. Psychoeducation on suicidal thoughts; symptom monitoring; safety plan; digital hope kit; list of self-help skills + Treatment as usual	Treatment as usual in a specialized outpatient suicide prevention clinic (RCT)	~ 9.4	60, 33.3%	NR	Adults referred to treatment or evaluation for to suicidal thoughts	• ↓ in suicide risk in app group < control (only at trend level at 4-month follow up) • No between-group differences in depression	56.7% (34)
[49]	**BackUp**.^a^ Hope box (see VHB); coping cards; tools to recognize warning signs, use coping strategies, and reach out to others	None	1	21, 23.9%	NR	Adults with suicidal thoughts as measured by BSS	• Non-significant ↓ in SI	4.76% (1)
**Emotion Regulation Applications**								
[38]	**RELAX**. Tracks anger frequency, intensity, and cues; personalized anger management exercises + Anger management training group	Twice-weekly anger management training outpatient group alone (RCT)	6	28, 100%	NR	Adult armed forces veterans with high levels of STAXI-measured anger	• No differences between app group and control • ↓ anger severity and PTSD in both groups	10.7% (3)
[41]	**Headspace**. Brief mindfulness meditation exercises + acute state hospital treatment.	None	1	12, 83%	NR	Adults with schizophrenia or bipolar disorder and history of aggression or violence.	• No change in state or trait anger	16.7% (2)
[45]	**RELAX**. See above + Anger management training group	None	6	4	NR	Adult armed forces veterans participating in anger management group.	• ↓ in anger, PTSD and depression symptoms at post-tx and 3-month follow-up	25.0% (1)
[17]	**EMOTEO**. Provides guided mindfulness and distress tolerance exercises depending on stated level of tension + specialized BPD treatment	None	24	16, 0%	100%	Adult women with BPD in specialized treatment	• ↓ aversive tension	NR
**Multipurpose (Suicide/Self-Harm, Impulsivity, & Emotion Regulation) Applications**								
[7]	**VHB**. Repository of tools for distraction, relaxation, & stress-coping; fond memories; inspirational quotes + Treatment as usual	Printed materials about coping with suicidality + Treatment as usual (RCT)	12	50, 62.1%	NR	Adult armed forces veterans in treatment with current/past-3-month SI	• Stress coping in VHB > TAU group • No between-group differences in SI, reasons for living, suicide severity, and perceived stress	13.8% (8)
[54]	**DBT Coach**. Suggests emotion-specific opposite action behaviors, tracks emotional intensity and urges to use substances + DBT	None	2	22, 18.2%	100%	Adults with BPD and SUD in current DBT treatment	• ↓ in emotional intensity and urges to use substances • ↓ in depression and general distress	0% (0)
[55]	**DBT Coach**. Same as above but expanded to include most DBT skills, tracks distress and urges to self-harm + DBT	None	36	14, 25%	100%	Adults with BPD and recent history of NSSI and/or suicide attempts	• ↑ app use predicted larger ↓ in NSSI, but not psychopathology, suicidal behavior, or skills use	25% (4)
[67]	**Ibobbly**.^a^ Psychoeducation on thoughts, strategies to defuse them and regulate emotions; action plans for value-based goals	Waitlist (RCT)	6	31, 35.5%	NR	Indigenous Australian adults with past-2-week suicidal thoughts	• No between-group differences in SI, impulsivity • ↓ in depression and distress in app group > waitlist	6.45% (2)

*BPD* Borderline personality disorder; *DBT* Dialectical behavior therapy, *NR* Not reported, *NSSI* Nonsuicidal self-injury, *PTSD* Post-traumatic stress disorder, *RCT* Randomized controlled trial, *RELAX* Remote Exercises for Learning Anger & Excitation Management, *SI* Suicidal ideation, *SITB* Self-injurious thoughts and behaviors, *STAXI* State-trait anger expression inventory, *SUD* Substance use disorder, *TAU* Treatment as usual, *VHB* Virtual Hope Box

^a^= standalone intervention

^b^= data presentation not amenable to meta-analysis

^c^Computed from received allocated intervention/accessed the smartphone application, from intervention condition

↓ = decrease

↑ = increase

In terms of therapeutic approach, a significant majority (*N* = 9) of smartphone applications were based at least in part on principles of cognitive-behavioral therapy (CBT) [7, 18, 38, 40, 45, 47, 49, 52, 54, 55, 67] and one was based on principles of mindfulness [41]. The CBT-based applications suggested skills and strategies to use when faced with intense emotions. One exception was TecTec, which instead employed behavioral conditioning to modify positive and negative associations with the self and self-harm [18]. Other common features included psychoeducation, delivered in Life App’Tite and ibobbly [47, 67]; symptom monitoring, which was utilized by RELAX, Life App’tite, EMOTEO, and DBT Coach [38, 45, 47, 52, 54, 55]; and safety plans, which were used in BeyondNow, Life App’tite, and BackUp [40, 47, 49].

In terms of symptom clusters targeted, 6 of the 10 smartphone applications targeted behavioral dyscontrol symptoms commonly occurring in BPD. DBT Coach and ibobbly addressed impulsivity [54, 55, 67], while also addressing nonsuicidal self-injury or suicide attempts along with Virtual Hope Box, BeyondNow, Life App’tite, and BackUp [7, 40, 47, 49]. Five applications targeted emotional symptoms, specifically Virtual Hope Box, EMOTEO, and DBT Coach for affective instability [7, 52, 54, 55], and RELAX and Headspace for anger specifically [38, 41, 45]. None targeted symptoms related to interpersonal sensitivity, distorted cognition, or identity disturbance.

Of the 7 RCTs reported in these 5 articles, 5 reported significant improvements: in depression and distress in the ibobbly group over the six-week follow-up [67], in self-harm in the TecTec group across the 3 trials [18], and in stress coping in the VHB group [7] compared to their control groups. In contrast, Mackintosh et al. [38] found no differences between the RELAX group and the control group over the six-month posttreatment follow-up, while O'Toole et al. [47] actually found a *smaller* decrease in suicide risk in the Life App’tite compared to the control group, although this was only at the trend level at the four-month follow-up. In addition, the positive effects of TecTec were not maintained 1 month posttreatment [18].

Of the 7 pre-post trials, 6 reported generally favorable results. There were significant improvements in emotional intensity and urges to use substances [54] and self-harm for DBT Coach users over the three-month follow-up [55]; in suicide-related coping, severity and intensity of suicidal ideation for BeyondNow users [40]; and in aversive tension for EMOTEO users [52]. Another two studies reported nonsignificant trends: BackUp resulted in decreases in suicidal ideation [49], and RELAX resulted in reductions in anger, and posttraumatic stress disorder and depression symptoms in the 4 users over the three-month follow-up [45]. Mistler et al. [41] reported no significant change in anger following use of Headspace. No posttreatment follow-up data was available for BeyondNow, Headspace, BackUp, EMOTEO, and the first version of DBT Coach [40, 41, 49, 52, 54].

Most included studies did not report serious adverse events. There was one attempted suicide during the trial of BeyondNow, and multiple during the trials of TecTec [18, 40]. Both the Life App’tite trial and one of the three TecTec trials reported a slower reduction in suicidality (i.e., suicide risk and suicidal ideation and plans, respectively) in the group that used the application compared to the control group post-treatment [18, 47].

#### Availability and usability

The studies reported promising data on smartphone application usage, with available rates of usage ranging from 70.2–100%, with “usage” defined as accessing the application at least once. Dropout rates ranged from 0 to 56.7% (*M* = 22.5, 95% CI [12.4, 32.6]), with “dropout” computed as the percentage of participants receiving the intervention who did not complete the final follow-up, or in the case of Rizvi et al. [55], dropped out of treatment.

#### Commercially available applications

Six smartphone applications were available at no cost in both the App Store and Google Play: VHB [7], TecTec [18], Headspace [41], BackUp [49], EMOTEO [52], and ibobbly [67]. The median rating was 5 out of 5 stars, although in most cases the number of raters was low (*N* < 35). Headspace had the greatest number of downloads at 10,000,000+, and was rated 3.7–4.9/5 by users (*N* = 702,229 ratings), followed by VHB with 100,000+ downloads and a 4/5 rating by users (*N* = 962 ratings). BackUp and EMOTEO both had over 5000 downloads, but BackUp was rated higher at 3.7–5 out of 5 (*N* = 26 ratings) than EMOTEO, which was rated 3.3 (*N* = 31 ratings). BackUp’s number of downloads is also notable considering it is only available in Flemish – and the only application to use a language other than English. ibobbly had the lowest number of downloads (50+), but was rated 5 out of 5 by 3 users. Rating and download data was unavailable for TecTec.

In general, the App Store and Google Play data show variable engagement in these applications, which aligns with the usability data reported in the articles. The minimum reported percentage of participants that accessed the smartphone application at least once during the study period was 70% [18], with other studies citing higher participation rates. The studies that provided data on frequencies of use reported 76.1% (*N* = 16) of BackUp users accessing it at least several times [49], 67% (*N* = 8) of Headspace users using it “often” [41], 56.9% of VHB users (*N* = 33) accessing it at least a few times a week [7], and EMOTEO users accessing it an average of 1.71 (SD = 3.62) times per day over the course of the 6 month trial [52].

Three studies asked participants about helpfulness and likelihood of using the smartphone application in their daily lives. Bush et al. [7] reported lower percentages, with 70.1% of VHB users rating it as at least somewhat helpful and 55.2% indicating they were likely to use it again. Sixty-seven percent of Headspace users thought it was helpful in managing their symptoms and endorsed willingness to use it in the future [41], and 80% of BackUp users rated the application as helpful and 70% said they would use it in daily life [49].

All applications are available at no cost and most authors do not declare competing interests, aside from Franklin et al. [18]. The first author J. C. Franklin owns Tec-Tec, LLC (limited liability company), and at the time of publication had a pending patent application with the third author, C. R. Franklin. VHB [7] was made available by the National Center for Telehealth and Technology, part of the U.S. Military Health System. While Headspace, Inc. provided free use of their product to Mistler et al. [41], they were not involved in the conduct, analysis or reporting of the research, and no study authors had any type of financial relationship with them. BackUp [49] was funded by the Flemish government, ibobbly [67] was funded by the Australian Government Department of Health and Aging, and EMOTEO [52] was supported by funds from and is made available by The University Hospitals of Geneva.

#### Commercially unavailable applications

The other four applications (BeyondNow, DBT Coach, Life App’Tite, and RELAX) could not be found on the App Store or Google Play. Nevertheless, the studies that investigated their effectiveness reported somewhat favorable data on their usability. The minimum reported percentage of participants that accessed the smartphone application at least once during the study was 77.3% [40], and qualitatively reported strengths of these applications included portability, discreetness, and customizability [45], ease of use [38, 45], and the provision of hope, connection and utility [40].

Three studies asked participants about helpfulness, and 2 asked about the likelihood of using the smartphone application in their daily lives. Mackintosh et al. [38] and Morland et al. [45] reported helpfulness rating of 4.5–5 out of 6 for RELAX, while participants of Rizvi et al. [54] rated DBT Coach’s opposite action coaching helpful 96.8% of the time. Moreover, the 2 trials for DBT Coach reported that 90–100% of participants would use the application of their own initiative [54, 55].

That said, Rizvi et al. [55] found that DBT Coach users gave low ratings on how enjoyable and interesting it was, and Mackintosh et al. [38] reported that RELAX users spent a significantly shorter time practicing skills between sessions compared to the group that received anger management treatment alone. The usability of these applications may therefore be limited or have unintended consequences for skills practice and consolidation.

### Risk of Bias

**Fig. 2:**
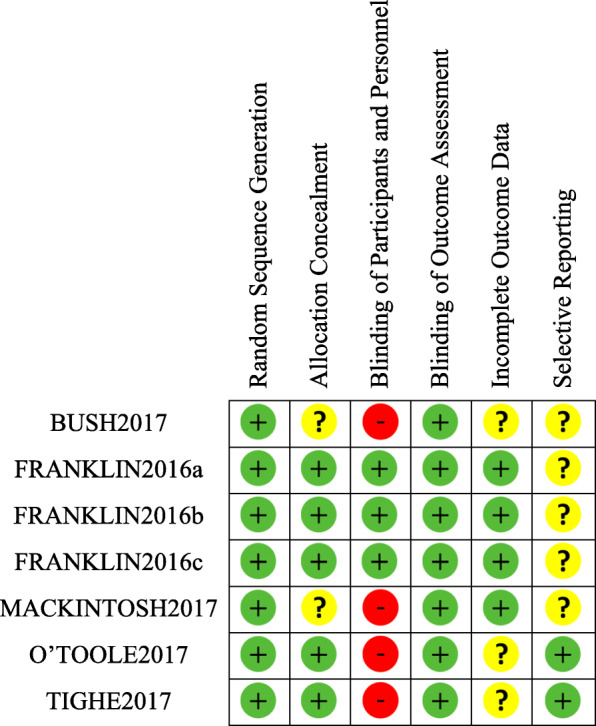
Risk of bias summary

Risk of bias per study is presented in Fig. 2. Risk of bias assessment revealed low risk of bias in the included RCTs in areas of random sequence generation and blinding of outcome assessment. Allocation concealment was unclear in 2 studies but presented low risk of bias in 5. For most studies, it was impossible to conceal allocation due to the near impossibility of masking psychological interventions, except for the RCTs reported by Franklin et al. [18], which presented participants in both arms with automated therapeutic evaluative conditioning (TEC) tasks with minor differences that were unlikely to be apparent to participants. For 3 studies, it was unclear whether attrition bias (incomplete data reporting) significantly affected the results, whereas for 4, risk of this form of bias was deemed low. Only 2 studies had a study protocol available that made it possible to determine they were at low risk of bias for selective reporting, while the remaining 5 did not. Studies that reported more positive effect sizes [18, 67] did not have different risk of bias profiles from other studies in this analysis with more unfavorable outcomes [18, 47]. Meta-regression analysis found no significant moderation of effect sizes by the number of risk of bias criteria rated low.

### Quantitative review

#### Between-groups meta-analysis of RCTs

**Fig. 3:**
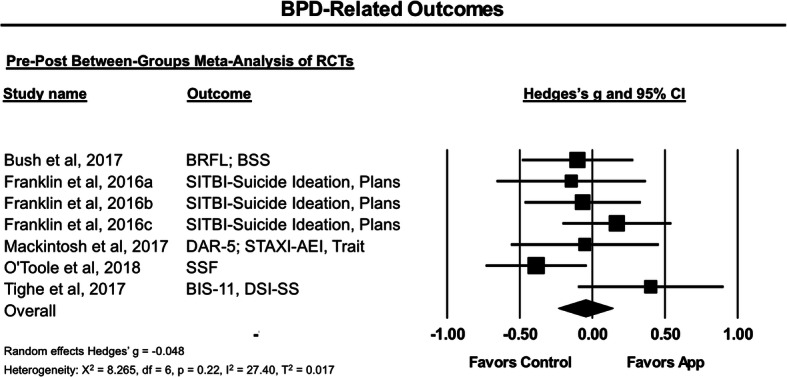
Forest plot of effect of smartphone applications on borderline personality disorder (BPD) symptoms. BIS-11 - Barratt Impulsiveness Scale-11 item; BRFL - Brief Reasons for Living Inventory; BSS - Beck Scale for Suicidal Ideation; DAR-5 - Dimensions of Anger Reactions-5; DSI-SS - Depressive Symptom Inventory-Suicidality Subscale; SITBI - Self-Injurious Thoughts and Behaviors Interview; SSF-II-R - Suicide Status Form II-Revised

Five articles describing 7 RCTs reported between-groups data on the primary outcome of BPD-related symptoms using 7 measures of suicidality and NSSI, 2 measures of anger, and 1 measure of impulsivity, as listed in Table 2. A Hedges’ *g* of −.05 (95% CI [−.24, .14]) was not statistically significant, *I*^*2*^ = 27.40%, *p* = .62 (Fig. 3).

**Table 2:** Outcome measures and groupings used in meta-analysis

Study	App of Interest	N	Control Condition	N	Length (weeks)	Outcome measures included in meta-analysis	
						BPD-related outcomes	General psycho-pathology
[7]	Virtual Hope Box (+TAU)	50	EUC + TAU	55	12	BRFL, BSS	PSS
[18]	Tec Tec	33	Control TEC with neutral pictures	25	4	SITBI	
[18]		44		52	4		
[18]		51		58	4		
[38]	RELAX (+AMT)	28	AMT	30	6	DAR-5, STAXI	PHQ-9
[47]	LifeApp’tite (+TAU)	60	TAU	69	~ 9.4	SSF-II-R	MDI
[67]	Ibobbly	31	Waitlist	30	6	DSI-SS; BIS-11	PHQ-9, K10

*AMT* Anger Management Treatment, *BIS-11* Barratt Impulsiveness Scale-11 item, *BRFL* Brief Reasons for Living Inventory, *BSS* Beck Scale for Suicidal Ideation, *DAR-5* Dimensions of Anger Reactions-5, *DSI-SS* Depressive Symptom Inventory-Suicidality Subscale, *EUC* Enhanced Usual Care, *K10* Kessler Psychological Distress Scale-10-item, *MDI* Major Depression Inventory, *PHQ-9* Patient Health Questionnaire-9, *PSS* Perceived Stress Scale, *SITBI* Self-Injurious Thoughts and Behaviors Interview, *SSF-II-R* Suicide Status Form II-Revised, *STAXI* State-Trait Anger Expression Inventory, *TAU* Treatment as Usual

Data from the RCTs also suggested these smartphone applications were not associated with a significant effect on general psychopathology, as measured by 2 measures of depression, a measure of stress, and a measure of distress across 4 trials. A Hedges *g* of .31 (95% CI [−.14, .75]) was not significant and there was substantial heterogeneity, *I*^*2*^ = 76.16%, *p* = .18.

### Publication Bias

Inspection of funnel plots and Duval and Tweedie’s trim and fill procedure [11] revealed no publication bias for BPD symptoms, nor for general psychopathology outcomes. Egger’s intercept [13] failed to reach significance across outcomes, again suggesting no publication bias.

## Discussion

We systematically reviewed 10 smartphone applications designed as interventions for BPD-related symptoms, and performed a meta-analysis for the 7 RCTs. This meta-analysis revealed no significant differences in effects of conditions with and without smartphone applications on BPD-related symptoms or general psychopathology. Symptom targets included state dimensions of BPD, including self-harm, suicidality, emotion dysregulation, anger, and impulsivity. The included studies were conducted in North America, Europe, and Australia, with limited data from other parts of the world, and limited data in general as the literature on smartphone applications for BPD symptoms is scarce.

These findings, based on a small group of studies, suggest that BPD-related interventions delivered via smartphone applications are still incipient and it is too early to recommend them as standalone or even adjunctive treatments. The qualitative synthesis revealed mixed results. While HeadSpace, Life App’tite, and BeyondNow evidenced no significant clinical contributions, the uncontrolled studies generally reported hopeful findings, with BeyondNow, RELAX, EMOTEO, and DBT Coach resulting in some reductions in BPD-related symptomatology. However, when smartphone applications were tested under more stringent conditions in controlled trials, there were rarely between-group differences in clinically relevant outcomes. The exceptions were the applications tested against low-intensity comparators, i.e. TecTec, which resulted in short-term improvements in NSSI compared to a control app; and iBobbly, which resulted in reduced depression and distress compared to a waitlist. The meta-analysis pooled outcomes of controlled studies, and found no evidence that smartphone applications confer any additional benefit in reducing BPD-related symptoms above and beyond a waitlist or the in-person treatments they were delivered alongside. While some of the study-level results are encouraging, the effect sizes found in this preliminary meta-analysis suggest that it is too early to make treatment recommendations involving smartphone apps for BPD-related symptoms or to use them in the place of existing treatments.

And yet, the development of resource-efficient treatment resources for BPD is critical to increase access to care and technology is a promising avenue for doing so in mental health care at large [12, 34]. Our review suggests that the adaptation of a range of treatment approaches to smartphone applications is user-friendly based on the ratings, number of downloads, frequency of use and interest in using the application beyond the study duration. The question, then, is how to capitalize on these user-friendly smartphone applications to contribute to efforts to increase resources for BPD care.

Given that there is no evidence that smartphone applications are helpful in reducing BPD-related symptomatology, but only 1 in 5 individuals with BPD are likely to seek live treatment from a psychiatrist or psychologist, the field needs more data on what specific roles these smartphone applications might occupy in the treatment landscape of BPD. One possible role is to provide psychoeducation or skills training to individuals with BPD symptoms. In terms of purpose and content, the majority of smartphone applications considered in our analyses were (a) adjunctive and (b) were based on CBT principles and DBT skills. Since the therapeutic approach most often represented in the design of the applications in this study, as well as other reviews [37], was CBT and its variants, this suggests CBT- and DBT-based interventions may prove better suited for technological interface than other evidence-based approaches (i.e. psychodynamic psychotherapies like MBT or TFP), possibly because the level of psychoeducation and teaching involved can be delivered without face-to-face interaction with a healthcare professional [72, 74]. Psychoeducation in and of itself has been shown to be helpful in reducing BPD symptoms, even when internet-delivered, so smartphone applications can at least be a potential avenue for equipping individuals with basic knowledge of the disorder [77].

A second potential role of smartphone applications would be to minimize phone coaching, as clinicians can find it difficult to implement this component of BPD treatments due to personal time and resources unavailable for calls outside of business hours [32]. If so, this may broaden implementation of evidence-based treatments for BPD. Among the included studies, only Rizvi et al. [54] investigated whether their smartphone application reduced coaching calls. They did not find evidence that the frequency of coaching calls received by the therapist decreased during the trial of DBT Coach, but whether other smartphone applications could provide alternatives to intersession contact or even enhance the focus of such contact deserves further study.

Another potential role of smartphone applications could be to engage individuals who have not yet entered treatment. The majority of the included studies investigated the utility of adding a smartphone application to in-person treatments, i.e., increasing intensity of treatments that typically have already demonstrated efficacy. In order to investigate the utility of smartphone apps in targeting symptoms of BPD when no other options are available, studies should ideally compare the efficacy of a smartphone intervention alone to a waitlist control group, as in Tighe et al. [67], or to a control app that similarly has no accompanying in-person intervention, as in Franklin et al. [18]. Tighe et al. [67], the only study to have a waitlist comparison group, found no between-group differences in suicidal ideation or impulsivity, but did find lower depression and distress in the group that used iBobbly. Instead of increasing intensity of in-person treatments with patients who are already motivated to be in treatment, it would be beneficial to study whether these smartphone interventions can provide at least reduction in distress in the vast majority of people with BPD who are unable or unmotivated to access in-person treatment. Individuals who do not seek psychological or psychiatric help may need a low-intensity starting point to motivate access to further treatment. A study of two single-session suicide-focused interventions with 93 non-treatment-engaged participants found that half of the participants went on to seek mental health services during a 3-month follow-up period [73]. Furthermore, individuals with suicidal ideation have variable levels of willingness to engage in face-to-face treatment. When suicide risk increases, willingness to seek face-to-face help appears to decrease but for the subgroup of emerging adults, willingness to seek help from informal online sources appears to increase [63]. These suggestions as to the potential of smartphone applications to reach non-treatment-engaged individuals are tentative. More focused avenues of research are needed to gain clarity on how exactly smartphone applications can contribute to closing the treatment gap, given their current inability to replace or supplement in-person treatments.

These studies would need to be weighed with ethical considerations and clearly report usability and safety. In terms of usability, the utility of smartphone applications is limited by user engagement [69]. That 70–100% of participants in the included studies accessed the smartphone application at least once and that 67–100% would use it beyond the study duration is promising, but discontinuing usage of applications is common, with about 25% of users abandoning applications after one use, and 68% abandoning them after only 10 uses [56]. For health-related applications specifically, 45.7% of users report discontinuing usage due to time required (44.5%), loss of interest (40.5%), hidden costs (36.1%), confusion (32.8%), and dislike of data being shared with friends (29% [31];). While dropout rates in the present group of studies averaged at 22.5, 95% CI [12.4, 32.6], on par with dropout rates from DBT treatment (28, 95% CI [23.6, 32.9] [9];), they showed much higher variance (0–56.7%) and ranges of engagement reported in this study were wide. Given the general bias in research studies towards interpreting user engagement ratings as positive [46], the actual likelihood of application utilization is unclear at this point in time and further study is needed. In terms of safety, factors that may influence the perceived usability and safety risk (e.g. privacy policies [57];) of smartphone applications designed as interventions should be explored as well. Some of the included studies reported suicide attempts [18, 40] or slower reduction of suicide risk compared to the control group [18, 47], and the dearth of research on the potential adverse effects of mobile mental health technologies must be addressed [60].

Other directions for future research include studies with standard, reliable measures of BPD symptoms (see [21] for list of potential measures); more follow-up data to investigate whether application usage and improvements are sustained; homogenous measures across studies to ease pooling and comparison of outcomes; exploration of effective applications’ mechanisms of change; and study samples outside North America, Europe, and Australia. There are also opportunities to integrate applications with passive data collection and ambulatory/ecological momentary assessment/intervention [61, 70], which may allow for more sophisticated treatment development. Given the large variability in dropout rates between applications (0–56.7%), predictors of dropout should be analyzed to optimize user experience and maximize retention, e.g. technological barriers have been shown to significantly predict dropout from an Internet-delivered DBT intervention [75].

There are several limitations to this review and the scope of generalization of its findings. First and foremost, the majority of the apps included in this review, although designed to address symptoms of suicidality, NSSI, anger, and impulsivity that frequently occur in BPD, were not designed specifically with BPD in mind. Few studies screened for BPD and only three out of 12 required a BPD diagnosis. A second limitation is the narrow scope of this review. There are a number of Internet-delivered interventions that can be accessed on one’s smartphone that were not included because they were not smartphone applications, even if they have similar attributes of being portable and accessible (e.g. [74]). Thirdly, it is debatable whether a sample of studies this small merits a meta-analysis, especially given the substantial heterogeneity of the included studies in the general psychopathology analysis. The results of the meta-analysis should therefore be regarded as preliminary. Fourthly, the review was not registered, since it was not originally conducted with publication in mind, and the authors wished to avoid post hoc registration. However, lack of prospective registration introduces risk of selective reporting bias [65]. Fifthly, we calculated effect sizes based on baseline and immediate post-treatment scores, although some studies had multiple time points. There are also limitations on the study level. For example, the risk profile of the included participants across studies was relatively low. While the cautious methodology of these studies understandably restricted the levels of risk in their samples, patients with BPD present with high comorbidity [20, 64], and many patients will have active suicidal ideation, current severe psychopathology, need for inpatient treatment, or substance use disorders. Finally, psychosocial functioning is an important outcome that many of these studies did not measure.

## Conclusions

Notwithstanding these limitations, this study provides an overview of available and studied smartphone interventions, finding that these interventions are user-friendly, but no more effective than their comparison conditions. The research on these applications is scarce, development of these applications is still in the early stages, and for now, gold standard evidence-based specialist and generalist treatments for BPD should remain the recommendation of choice. While smartphone applications and other computerized solutions have proven to be a promising avenue for bridging the mental healthcare gap in other diagnoses, evidence is lacking to recommend them for this purpose to target BPD symptoms in the absence of further studies. Access to smartphones currently exceeds access to mental healthcare, and smartphone applications hold potential to provide some form of help when other forms of care are simply unavailable. More research is needed to investigate how to design these smartphone applications to be effective in contributing to BPD-related care.

## Data Availability

The dataset supporting the conclusions of this article is available in the Harvard Dataverse repository, 10.7910/DVN/4HLCL3.

## Abbreviations

AMT: Anger management treatment
BPD: Borderline personality disorder
CBT: Cognitive-behavioral therapy
DBT: Dialectical behavior therapy
DSM-IV: Diagnostic and statistical manual of mental disorders-fourth edition
DSM-5: Diagnostic and statistical manual of mental disorders-fifth edition
MBT: Mentalization-based treatment
NCS-R: National comorbidity survey-replication
NESARC: National epidemiologic survey on alcohol and related conditions
RCT: Randomized controlled trial
RELAX: Remote exercises for learning anger & excitation management
SFT: Schema-focused therapy
SITB: Self-injurious thoughts and behaviors
TAU: Treatment as usual
TEC: Therapeutic evaluative conditioning
TFP: Transference-focused psychotherapy
VHB: Virtual hope box

## Footnotes

1 For borderline personality disorder, *borderline, borderline personality,* or *borderline personality disorder*. For criterion 1, *abandon**. For criterion 2, *interpersonal effectiveness, interpersonal sensitivity, interpersonal hypersensitivity, rejection sensitivity*. For criterion 3, *identity disturbance*. For criterion 4, *impuls**. For criterion 5, *non-suicidal self-injury, self-injur*, self-harm, parasuicid*, suicid**. For criterion 6, *emotion dysregulation, emotional dysregulation, affective instability, affective lability, emotion regulation, emotional regulation, mood instability, mood lability, aversive tension*. For criterion 7, *empt**. For criterion 8, *anger, angr**. For criterion 9, *depersonalization, depersonalisation, derealization, derealisation, dissociat*, paranoi**.
